# Influence of Final Irrigation on Calcium Silicate-Based Sealer Dentinal Tubular Penetration: A Systematic Review

**DOI:** 10.3390/ma19122682

**Published:** 2026-06-22

**Authors:** Jordi Gómez-González, Daniela Fernández-Negrete, José Luis Sanz, James Ghilotti, Sofía Folguera, Adrián Lozano

**Affiliations:** Department of Stomatology, Faculty of Medicine and Dentistry, Universitat de València, 46010 Valencia, Spain

**Keywords:** calcium silicate, endodontics, irrigation, in vitro, penetration, sealer, systematic review

## Abstract

The aim of this systematic review was to assess the influence of different final irrigation protocols and activation methods on the dentinal tubular penetration of calcium silicate-based sealers (CSSs). The review followed the PRISMA 2020 guidelines, and the protocol was registered in the Open Science Framework (OSF; DOI: 10.17605/OSF.IO/5HTVN). A PICOS-based research question was formulated, and a comprehensive literature search was conducted in MEDLINE, Scopus, Embase, Web of Science, and SciELO up to October 2025. After study selection, a qualitative synthesis of methodologies and outcomes was performed, and methodological quality was assessed using the QUIN tool. Twenty-one in vitro studies were included, all of which used single-rooted teeth or single roots. The available evidence suggests that final irrigation protocols may influence CSS penetration, although the magnitude and consistency of this effect varied substantially across studies. Sodium hypochlorite (NaOCl) and 17% EDTA were the most frequently investigated irrigants and were generally associated with improved penetration, but no irrigation protocol or activation technique can currently be considered superior. Current research trends include the evaluation of chelating agents, continuous chelation protocols, and irrigant activation systems such as passive ultrasonic irrigation, sonic activation, laser activation, and XP-Endo Finisher. Future studies should standardize irrigation protocols, activation methods, sealer types, obturation techniques, microscopy-based assessment procedures, and penetration outcome measures, while also including larger samples and more anatomically complex root canal systems.

## 1. Introduction

Irrigation constitutes one of the fundamental pillars of root canal treatment. The complex anatomy of root canal systems renders irrigation indispensable for the removal of organic tissue, dissolution of pulpal remnants, reduction in bacterial load, and facilitation of the proper action of endodontic sealers [1,2]. In endodontics, irrigation is defined as the process of introducing liquid solutions into the root canal system during root canal treatment with the aim of cleaning, disinfecting, and facilitating instrumentation. Irrigating solutions are delivered using needles, syringes, or activation systems, and they act in areas inaccessible to endodontic files due to the complex dental anatomy [3,4].

Various liquid solutions are used as irrigants in endodontics [3]: primary irrigants with antimicrobial activity (sodium hypochlorite (NaOCl) and chlorhexidine (CHX)); chelating irrigants for smear layer removal (17% EDTA, citric acid, and HEBP); irrigants with detergent or surfactant action (QMix); and other less frequently used irrigants (hydrogen peroxide, MTAD, and liquid ozone). The scientific literature also reports additional substances employed as irrigants during root canal treatment, including 7% maleic acid [5,6,7], glycolic acid [8,9,10], apple cider vinegar [11,12,13,14,15], chitosan nanoparticles [16], and peracetic acid [17].

The different methods used to activate irrigants during root canal treatment include the following [18,19]: manual activation; sonic activation; passive ultrasonic irrigation (PUI); rotary mechanical activation; apical negative pressure irrigation; and laser activation. Other irrigation activation techniques include heat activation [20] and lentulo spiral activation [21].

The most widely known endodontic cements or sealers used during root canal filling are as follows [22]: zinc oxide–eugenol (ZOE), resin-based sealers, calcium silicate-based sealers (CSSs), zinc phosphate, and calcium hydroxide-based sealers. An increasing number of published studies have investigated the use of CSSs for root canal filling, owing to their favorable characteristics, including biocompatibility, bioactivity, dentinal tubule penetration, and suitable physical properties. Nevertheless, their outcomes and performance continue to be evaluated in comparison with other types of sealers, such as resin-based sealers or ZOE [23].

To assess the penetration of endodontic sealers into dentinal tubules, high-resolution and/or fluorescence microscopy techniques are primarily employed [24]. The most used microscopy methods include confocal laser scanning microscopy (CLSM), scanning electron microscopy (SEM), fluorescence microscopy (FM), and micro-computed tomography (micro-CT). Among these, CLSM is the most frequently used technique [25]. A wide variety of image-processing software programs are utilized to analyze the acquired images, including proprietary microscope software as well as independent or complementary programs; however, ImageJ is the most used software [26].

To clearly differentiate sealer penetration within dentinal tubules, sealers are commonly mixed with fluorescent dyes that can be detected under CLSM. Rhodamine B has been widely used for this purpose in endodontic sealer penetration studies; however, concerns have been raised regarding dye leaching and possible overestimation of true sealer penetration, particularly when calcium silicate-based sealers are evaluated [27]. For this reason, alternative fluorescent markers, including calcium-sensitive dyes such as Fluo-3, have also been proposed and used in some studies [28,29]. Therefore, the interpretation of fluorescence-based penetration data should consider the type of dye used, its interaction with the sealer, and the possibility that fluorescence may not always correspond exactly to true material penetration.

When CLSM is used to analyze the penetration of endodontic sealers into dentinal tubules, several key variables are typically measured to quantify sealer efficacy [24]: penetration depth, percentage of tubule penetration, penetration area, three-dimensional penetration volume, fluorescence intensity, and penetration distribution or pattern. Lastly, another method described in the scientific literature to evaluate the effectiveness of sealer penetration into dentinal tubules is the push-out bond strength (PBS or POBS) test [30].

Calcium silicate-based sealers are increasingly used in root canal filling because of their biocompatibility, bioactivity, hydraulic setting behavior, and potential interaction with dentin. However, their dentinal tubular penetration may be affected by the chemical and physical conditions created by the final irrigation protocol, including smear layer removal, residual moisture, dentin surface changes, and the use of irrigant activation systems. Although numerous in vitro studies have assessed the effect of irrigants and activation methods on CSS penetration, their findings remain difficult to interpret collectively because of marked heterogeneity in tooth type, irrigation sequence, activation method, sealer formulation, filling technique, fluorescent dye, microscopy method, and penetration outcome parameters. Therefore, a systematic review was considered necessary to synthesize the available evidence, identify methodological trends and inconsistencies, and clarify the extent to which current in vitro data can support conclusions regarding the influence of final irrigation on CSS dentinal tubular penetration.

## 2. Materials and Methods

### 2.1. Protocol and Registration

The present work followed the guidelines recommended by the PRISMA 2020 Statement (Preferred Reporting Items for Systematic Reviews and Meta-analysis, details are provided in the Supplementary Materials) [31]. The protocol of the present systematic review was registered in Open Science Framework (OSF) registries with the following registration DOI: 10.17605/OSF.IO/5HTVN.

### 2.2. Search Strategy and Eligibility Criteria

A PICOS (Population, Intervention, Comparator, Outcome, Study design) question was formulated as follows: (P) Extracted teeth; (I) Root canal treatment performed using a specific irrigation method and root canal filling with CSSs; (C) An alternative irrigation method; (O) Dentinal tubular penetration or adaptation of the CSS; (S) In vitro study.

Accordingly, the research question developed to conduct the literature search was: How does the final irrigation method influence the penetration of calcium silicate-based sealer dentinal tubular penetration in extracted teeth subjected to root canal treatment?

To facilitate the search process, key terms derived from the PICO framework were selected to transform the research question into a search strategy. Controlled vocabulary terms, such as MeSH or Emtree terms, were not used to maintain consistency across all databases searched and to account for variability in indexing and terminology across the available in vitro literature. The literature search was conducted in October 2025 in the following electronic databases: MEDLINE, Scopus, Embase, Web of Science, and SciELO. The complete search strategy and findings per database are shown in Table 1.

Regarding the eligibility criteria, studies which met the established PICOS criteria, that is, in vitro studies on the dentinal tubular penetration of one or more calcium silicate-based sealers in extracted teeth undergoing root canal treatment with different irrigation methods, were considered as eligible.

### 2.3. Study Screening

The articles identified using the selected search terms were exported to the reference management software Mendeley (version 1.19.8; Elsevier, Amsterdam, The Netherlands) to remove duplicate records. After duplicate record removal, an initial screening of titles and abstracts was conducted according to the predefined eligibility criteria. Subsequently, a second screening was performed through full-text assessment of the remaining articles to confirm their eligibility. Study screening was performed by 2 reviewers (J.G.-G. and J.L.S.) independently. Disagreements were resolved by discussion and consensus with a third reviewer (A.L.).

### 2.4. Data Extraction

Data extraction was performed by 2 reviewers (J.G.-G. and A.L.). To extract the relevant data from the selected studies, standardized tables were developed including common variables to facilitate comparison. In the first table, methodological variables common to the included studies were recorded: author and year; type of tooth, sample size, and grouping criteria; instrumentation protocol; final irrigation protocol; sealer(s) used; and root canal filling technique. Subsequently, a second table was prepared to summarize outcome-related variables: author and year; method of penetration assessment (parameters evaluated); and main results.

### 2.5. Quality Assessment

To assess the methodological quality and risk of bias of the selected articles, the QUIN (Quality Assessment Tool for In Vitro Studies) tool was applied [32]. This instrument consists of a 12-item checklist designed to evaluate methodological rigor and study design in in vitro investigations conducted in the field of dentistry.

The methodological quality and risk of bias of the included studies were assessed by 2 reviewers (J.G.-G. and A.L.) using the QUIN tool. Each item was scored as 2 when adequately reported, 1 when inadequately reported or unclear, and 0 when not reported or associated with high risk of bias. The final percentage score was used to classify each study as low risk of bias (>70%), moderate risk of bias (50–70%), or high risk of bias (<50%). Individual item scores of 0 or 1 were interpreted as domain-specific methodological or reporting concerns and were considered in the qualitative interpretation of the evidence, even when the overall study score remained within the low-risk category.

## 3. Results

### 3.1. Study Selection and Flow Diagram

The initial literature search conducted in the databases identified a total of 82 potentially eligible articles, of which 12 were discarded as duplicates. Following the first screening of the title and abstract of the resulting records, 52 articles were excluded because they did not fulfil the eligibility criteria. The remaining 18 studies were deemed as eligible, and their full texts were retrieved.

A secondary search was subsequently performed by manually screening the reference lists of the selected articles. This process yielded 3 additional studies, for which full-text versions were also obtained. Ultimately, a total of 21 articles were included in the review [33,34,35,36,37,38,39,40,41,42,43,44,45,46,47,48,49,50,51,52,53].

**Figure 1:**
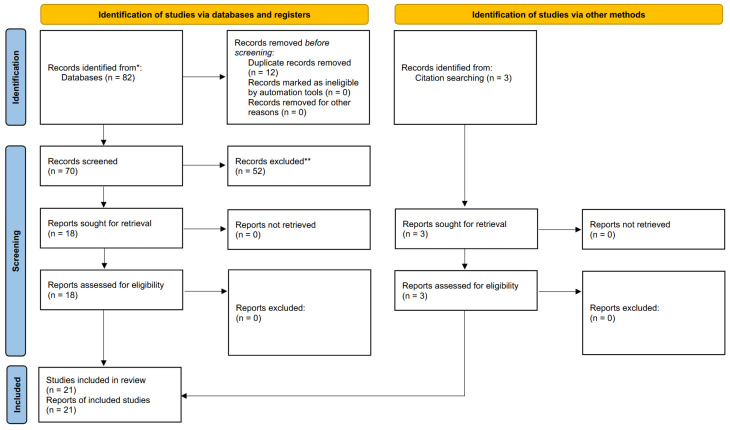
Study selection flow-chart. Based on the PRISMA flow diagram [28]. * Using the search strings from Table 1. ** Records screened in this phase were excluded for not fulfilling the eligibility criteria.

The study selection process is illustrated in Figure 1.

### 3.2. Quality Assessment

The results of the quality assessment of the in vitro studies are presented in Table 2, using the criteria of the QUIN (Quality Assessment Tool for in vitro Studies) [29] to calculate a quality score and a risk of bias. All the studies analyzed were rated as having a low risk of bias.

### 3.3. Study Methodology and Results

Extracted data regarding the included studies’ methodology and results are shown in Table 3 and Table 4, respectively.

## 4. Discussion

### 4.1. Interpretation of the Main Findings

The present systematic review synthesized the available in vitro evidence on the influence of final irrigation protocols and irrigant activation methods on the dentinal tubular penetration of calcium silicate-based sealers. Overall, the included studies suggest that final irrigation may affect CSS penetration; however, the magnitude and direction of this effect varied substantially across studies. This variability prevents the identification of a clearly superior irrigation protocol or activation method.

The studies that evaluated activation methods included conventional irrigation, passive ultrasonic irrigation, laser-assisted activation, sonic activation, XP-Endo Finisher, heat activation, and other activation approaches [33,36,38,39,44,49,51,52,53]. In general, activation methods tended to show better penetration outcomes than conventional irrigation, but the results were not consistent across studies. Some investigations reported better outcomes with laser activation, whereas others favored passive ultrasonic irrigation, XP-Endo Finisher, or other activation methods [33,36,38,39,44,49,51,52,53]. These inconsistencies are likely related to differences in activation devices, activation times, irrigant volumes, final irrigation sequences, sealer formulations, obturation techniques, and outcome assessment methods.

The studies that evaluated final irrigation protocols also showed substantial heterogeneity. The protocols included saline solution, EDTA, NaOCl combined with HEBP, maleic acid, glycolic acid, apple cider vinegar, chitosan nanoparticles, QMix, citric acid, and chlorhexidine [34,35,37,40,41,42,43,45,46,47,48,50]. Among these, EDTA and NaOCl-based protocols were the most frequently investigated and were often associated with favorable penetration results. However, this should not be interpreted as definitive evidence of superiority, because the included studies differed in the complete irrigation sequence, irrigant concentration, final rinse, canal drying protocol, sealer type, and penetration assessment method.

A recurrent trend across the included studies was that sealer penetration was generally greater in the coronal and middle thirds than in the apical third [33,34,36,37,39,42,45,47,50,51]. This finding may be explained by the anatomical and technical characteristics of the apical region, where dentinal tubule density, canal diameter, irrigant exchange, and smear layer removal may be less favorable. Nevertheless, this observation should be interpreted cautiously, because the studies used different penetration parameters, including maximum penetration depth, mean penetration depth, penetration area, penetration percentage, push-out bond strength, and other variables.

To facilitate interpretation of the heterogeneous findings, a concise summary of the main laboratory trends identified across the included studies is provided in Table 5. This table does not aim to establish the superiority of any specific irrigant or activation method, but rather to synthesize the overall direction of the evidence and highlight the main methodological limitations affecting comparison across studies.

### 4.2. Methodological Considerations

The interpretation of the available evidence is limited by the interaction of multiple methodological variables. The effect of a final irrigant cannot be isolated from the complete irrigation protocol in which it was used. For example, some studies used distilled water or saline after the final irrigant, whereas others did not apply an additional rinse. The use of distilled water as the sole final irrigant may reduce CSS penetration compared with protocols that remove the smear layer effectively, whereas its use after chelating agents may have a different effect depending on the overall sequence [54]. Therefore, the clinical or laboratory meaning of a given irrigant depends not only on the solution itself, but also on its concentration, sequence, activation, contact time, and final rinse.

Activation methods may also modify the effect of the irrigant by improving irrigant distribution, fluid movement, and smear layer removal. This may partly explain why some studies reported better outcomes when irrigants were activated, even when the same final irrigant was used [55]. However, activation protocols were not standardized across the included studies, and different systems were frequently tested with different sealers and different evaluation methods. Consequently, comparisons between activation systems should be interpreted as exploratory rather than definitive.

Moisture control is another relevant methodological factor when CSSs are evaluated. These materials require moisture for setting and may interact chemically with dentin. Therefore, residual moisture, canal drying procedures, and the use of paper points after final irrigation may influence their penetration, adaptation, and physicochemical behavior [56]. Differences in moisture control among studies may have contributed to the variability of the reported outcomes.

### 4.3. Risk of Bias and Overall Confidence of the Evidence

Although all included studies were classified as having a low risk of bias according to the global QUIN score, this finding should be interpreted cautiously. The final QUIN classification was based on the total percentage score obtained by each study; therefore, individual domains scored as “0” or “1” did not necessarily change the global category when the overall score remained above the predefined threshold. Consequently, a low overall QUIN classification should not be interpreted as absence of methodological or reporting concerns. Domain-level weaknesses, including incomplete reporting, unclear allocation procedures, variable sample sizes, and inconsistent outcome assessment methods, may still reduce confidence in the findings and should be considered when interpreting the body of evidence.

Lastly, to provide a more structured appraisal of the strength of the available evidence, overall confidence was considered according to study design, risk of bias, consistency, directness, precision, and comparability. The overall confidence in the evidence was judged to be limited. This judgment was based on the exclusive inclusion of in vitro studies, the use of dentinal tubular penetration as a laboratory surrogate outcome, substantial methodological heterogeneity, inconsistent penetration outcome measures, variable sample and group sizes, and the inability to perform quantitative synthesis. Therefore, the findings should be interpreted as general laboratory trends rather than high-certainty evidence supporting the superiority of any specific irrigation protocol, chelating agent, or activation method.

### 4.4. Clinical Implications and Limitations

From a practical perspective, the available evidence supports the importance of final irrigation protocols that promote smear layer removal and appropriate dentin surface conditioning before obturation with CSSs. NaOCl and EDTA remain the most frequently investigated irrigants, and irrigant activation may be considered an adjunctive strategy to improve irrigant distribution and sealer penetration under laboratory conditions. However, the present review does not support recommending one specific irrigant, chelating agent, or activation system as superior.

Several limitations should be considered. First, all included studies evaluated in vitro outcomes, and no clinical outcomes were assessed. Dentinal tubule penetration is a laboratory parameter that may provide information about material distribution within dentin, but it should not be considered a direct surrogate for clinical success. Previous studies have reported no significant correlation between sealer penetration into dentinal tubules and sealability, as well as no correlation between sealer penetration and interfacial adaptation to root dentine [57,58]. Therefore, the findings of the present review cannot be directly extrapolated to treatment success, periapical healing, postoperative symptoms, or long-term clinical performance.

Second, all included studies used single-rooted teeth or single roots, which limits the applicability of the findings to multi-rooted teeth and more complex root canal systems. Third, sample sizes varied considerably, and in some studies the division of specimens into multiple experimental groups reduced the number of samples per group. Fourth, the included studies differed in tooth type, instrumentation protocol, irrigation sequence, activation method, sealer formulation, obturation technique, fluorescent dye, microscopy system, image-analysis software, and penetration outcome parameters. These factors reduce comparability and prevent definitive conclusions.

Lastly, the search strategy was based on free-text terms and database-specific syntax, without the use of controlled vocabulary terms such as MeSH or Emtree. Although the strategy was intentionally broad and sensitivity-oriented to account for heterogeneous terminology in the field, the absence of controlled vocabulary may have limited retrieval of some indexed records. This methodological limitation should be considered when interpreting the comprehensiveness of the search.

### 4.5. Future Perspectives

Future studies should aim to reduce methodological heterogeneity and improve reproducibility. Larger sample sizes, standardized instrumentation protocols, clearly defined final irrigation sequences, controlled activation parameters, standardized canal drying procedures, and consistent obturation techniques are recommended. In addition, future investigations should include multi-rooted teeth and more anatomically complex root canal systems to better reflect clinical conditions.

Outcome assessment should also be standardized. Future studies should clearly define which penetration parameters are being measured and should report microscopy settings, image-analysis procedures, dye selection, examiner calibration, and reproducibility of measurements. The use of reporting guidelines for in vitro dental research and laboratory studies in Endodontology, such as the PRILE 2021 guidelines, may help improve transparency, reproducibility, and methodological completeness [59,60,61]. The PRILE checklist and explanation/elaboration document may be particularly useful for standardizing the reporting of sample selection, specimen preparation, intervention protocols, outcome assessment, image analysis, and statistical methods in future laboratory-based endodontic studies [60,61].

Finally, further research should clarify whether the laboratory differences observed in dentinal tubular penetration have any meaningful relationship with clinically relevant outcomes. Until such evidence is available, the findings of in vitro studies should be interpreted cautiously and used to guide laboratory understanding rather than to establish definitive clinical recommendations.

## 5. Conclusions

The available in vitro evidence suggests that final irrigation protocols may influence the dentinal tubular penetration of CSSs, particularly when they promote smear layer removal and adequate dentin surface conditioning. NaOCl- and EDTA-based protocols were the most frequently investigated and were commonly associated with favorable penetration outcomes; however, due to substantial methodological heterogeneity, no specific irrigant, chelating agent, or activation technique can currently be considered superior. Clinically, these findings support the use of well-controlled final irrigation protocols, while emphasizing that dentinal tubular penetration remains a laboratory outcome and should not be directly extrapolated to clinical success. Future research should focus on standardized methodologies, more complex root canal anatomies, and clinically relevant outcomes.

## Figures and Tables

**Table 1:** Search strategy and findings per database.

Database	Search String	Results
Medline	**#1 All fields** (hypochlorite) OR (maleic acid) OR (EDTA) OR (chlorhexidine) OR (CHX) OR (hypochlorus acid) OR (serum) OR (saline solution) OR (citric acid) OR (MTAD) OR (Qmix) OR (irrig*)	159,072
	**#2 All fields** (bioceramic*) OR (silicat*)	35,969
	**#3 All fields** (seal*)	73,877
	**#4 All fields** (endodontic treatment) OR (root canal treatment) OR (root obturation)	48,472
	**#5 All fields** (penetration) OR (adaptation)	1,1182,358
	**#1 AND #2 AND #3 AND #4 AND #5**	**48**
Scopus	**#1 TITLE-ABS-KEY** (hypochlorite) OR (maleic acid) OR (EDTA) OR (chlorhexidine) OR (CHX) OR (hypochlorus acid) OR (serum) OR (saline solution) OR (citric acid) OR (MTAD) OR (Qmix) OR (irrig*)	6,259,870
	**#2 TITLE-ABS-KEY** (bioceramic*) OR (silicat*)	764,608
	**#3 TITLE-ABS-KEY** (seal*)	1,090,666
	**#4 TITLE-ABS-KEY** (endodontic treatment) OR (root canal treatment) OR (root obturation)	109,580
	**#5 TITLE-ABS-KEY** (penetration) OR (adaptation)	4,684,076
	**#1 AND #2 AND #3 AND #4 AND #5**	**3**
Embase	**#1** (hypochlorite) OR (maleic acid) OR (EDTA) OR (chlorhexidine) OR (CHX) OR (hypochlorus acid) OR (serum) OR (saline solution) OR (citric acid) OR (MTAD) OR (Qmix) OR (irrig*)	2,451,079
	**#2** (bioceramic*) OR (silicat*)	37,352
	**#3** (seal*)	99,303
	**#4** (endodontic treatment) OR (root canal treatment) OR (root obturation)	28,058
	**#5** (penetration) OR (adaptation)	711,005
	**#1 AND #2 AND #3 AND #4 AND #5**	**32**
Web of Science	**#1 TS=** (hypochlorite) OR (maleic acid) OR (EDTA) OR (chlorhexidine) OR (CHX) OR (hypochlorus acid) OR (serum) OR (saline solution) OR (citric acid) OR (MTAD) OR (Qmix) OR (irrig*)	1,999,574
	**#2 TS=** (bioceramic*) OR (silicat*)	163,874
	**#3 TS=** (seal*)	181,339
	**#4 TS=** (endodontic treatment) OR (root canal treatment) OR (root obturation)	17,303
	**#5 TS=** (penetration) OR (adaptation)	1,005,808
	**#1 AND #2 AND #3 AND #4 AND #5**	**37**
Scielo	**#1** (hypochlorite) OR (maleic acid) OR (EDTA) OR (chlorhexidine) OR (CHX) OR (hypochlorus acid) OR (serum) OR (saline solution) OR (citric acid) OR (MTAD) OR (Qmix) OR (irrig*)	71,694
	**#2** (bioceramic*) OR (silicat*)	1204
	**#3** (seal*)	2297
	**#4** (endodontic treatment) OR (root canal treatment) OR (root obturation)	814
	**#5** (penetration) OR (adaptation)	16,042
	**#1 AND #2 AND #3 AND #4 AND #5**	**0**

**Table 2:** Quality assessment results.

Article	Item 1	Item 2	Item 3	Item 4	Item 5	Item 6	Item 7	Item 8	Item 9	Item 10	Item 11	Item 12	Final	Risk of Bias
Ackay et al. (2016) [33]	2	0	2	2	2	1	2	2	2	1	2	2	83.34%	Low
Alim Uysal et al. (2021) [34]	2	0	1	2	2	1	2	2	2	1	2	2	79.17%	Low
Alnoury et al. (2024) [35]	2	1	2	2	2	1	2	2	2	1	2	2	87.5%	Low
Ates et al. (2021) [36]	2	0	1	2	2	1	2	2	2	1	2	2	79.17%	Low
Aydin et al. (2018) [37]	2	0	2	2	2	1	2	2	2	1	2	2	83.34%	Low
Bogari et al. (2022) [38]	2	0	2	2	2	1	2	2	2	1	2	2	83.34%	Low
El Hachem et al. (2018) [39]	2	0	1	2	2	1	1	2	2	1	2	2	75%	Low
Gawdat and Bedier (2022) [34]	2	2	2	2	2	1	2	2	2	1	2	2	91.67%	Low
Gupta et al. (2024) [42]	2	0	1	2	2	1	2	1	2	1	1	2	70.83%	Low
Hassan and Roshdy (2023) [42]	2	2	2	2	2	1	2	2	2	1	2	2	91.67%	Low
Lemos et al. (2022) [43]	2	0	2	2	2	1	2	1	2	1	0	2	70.83%	Low
Mahdi and Talabani (2025) [44]	2	2	2	2	2	1	1	2	2	1	2	2	87.5%	Low
Martinho et al. (2020) [45]	2	0	1	2	2	1	1	2	2	1	2	2	75%	Low
Mehdat et al. (2022) [46]	2	1	2	2	2	1	1	1	2	1	2	2	79.17%	Low
Öztürk et al. (2025) [47]	2	2	1	2	2	1	2	2	2	1	2	2	87.5%	Low
Razmi et al. (2016) [48]	2	0	1	2	2	1	1	2	2	1	2	2	75%	Low
Saghebi et al. (2025) [49]	2	2	2	2	2	1	1	2	2	1	2	2	87.5%	Low
Shekhar et al. (2025) [50]	2	1	2	2	2	1	1	2	2	1	2	2	83.34%	Low
Talreja et al. (2023) [51]	2	0	1	2	2	1	1	2	2	1	1	2	70.83%	Low
Vivek et al. (2025) [52]	2	0	1	2	2	1	1	2	2	1	2	2	75%	Low
Zand et al. (2019) [53]	2	0	2	2	2	1	2	2	2	1	2	2	83.34%	Low

2 = Adequately reported/low risk of bias, 1 = Inadequately reported/unclear risk, 0 = Not reported/high risk of bias.

**Table 3:** Study methodology.

Author (Year)	Teeth (*n*)	Instrumentation	Final Irrigation	Sealer (Dye)	Obturation
Ackay et al. (2016) [33]	Mandibular premolar (156: 39 × 4)	Sealer ^1^//irrigant activation ^2^ K10 + ProTaper (F4)	5% NaOCl + EDTA + 5% NaOCl; CI (conventional), PIPS (laser), PUI (passive ultrasonic)	AH Plus (39), iRoot SP (39), MTA Fillapex (39), Guttaflow Bioseal (39) (+rhodamine B)	ProTaper single cone
Alim Uysal et al. (2021) [34]	Single-rooted (84: 21 × 4)	Final irrigation ^3^ K10 + ProTaper Next (X3)	Saline//17% EDTA//7% MA//2.5% NaOCl + 9% HEBP	AH Plus//MTA Fillapex//EndoSequence (+rhodamine B)	Single cone (X3)
Alnoury et al. (2024) [35]	Mandibular single-rooted premolar (80: 20 × 4)	Final irrigation ^3^ ProTaper (F5)	Final irrigation + 5 mL saline; 17% GA; 5% apple vinegar; 17% EDTA; saline	Cerafill BC; EndoSequence BC	Gutta-percha single cone
Ates et al. (2021) [36]	Mandibular single-rooted incisor (64: 16 × 4)	Irrigant activation ^2^ K10 + EndoSequence Xpress	17% EDTA + 5.25% NaOCl (1 min) + distilled water; CEN (conventional), EA (EndoActivator), laser (YSGG), XPEF (XP-Endo Finisher)	EndoSequence (+rhodamine B)	EndoSequence gutta-percha (technique not specified)
Aydin et al. (2018) [37]	Mandibular premolar (60: 20 × 3)	Final irrigation ^3^ K10 + ProTaper Next (#40)	CNPs; QMix; 17% EDTA (+ distilled water)	TotalFill BC (+rhodamine B)	Gutta-percha single cone
Bogari et al. (2022) [38]	Single-rooted tooth (45: 15 × 3)	Irrigant activation ^2^ K15 + ProTaper Next (X3)	2 mL 5.25% NaOCl + 17% EDTA + H_2_O; G-1: conventional, G-2: ultrasonic, G-3: laser	TotalFill BC	Gutta-percha single cone
El Hachem et al. (2018) [39]	Maxillary central single-rooted incisor (50: 25 × 2)	Irrigant activation ^2^ K10 + K15 + ProTaper Universal (F4)	5.25% NaOCl + EDTA + NaOCl + H_2_O; CN: conventional, EA: EndoActivator	Novel Tricalcium Silicate Sealer (+rhodamine B)	Gutta-percha single cone
Gawdat et Bedier (2022) [34]	Mandibular premolar with one canal (42: 12 × 3)	Final irrigation ^3^ K15 + ProTaper Next (X4)	G-1: 2.6% NaOCl + H_2_O; G-2: DualRinse HEBP + H_2_O; G-3: 2.6% NaOCl + EDTA + H_2_O	Well-Root ST (+rhodamine B)	ProTaper Next X4 gutta-percha single cone
Gupta et al. (2024) [42]	Permanent single-rooted tooth (60: 20 × 3)	Final irrigation ^3^ K15 + ProTaper Gold (F3) + H_2_O after final irrigation	G-1: EDTA + 3% NaOCl + saline; G-2: 9% HEBP + 3% NaOCl; G-3: 10% citric acid + 3% NaOCl + saline	Bio-C (+rhodamine B)	Lateral condensation + temporary filling (CavitG)
Hassan et Roshdy (2023) [42]	Mandibular single-rooted premolar (20: 10 × 2)	Chelating agent ^4^ WaveOne Gold Glider (alternating medium and large)	G-1: 5.25% NaOCl + 17% EDTA; G-2: NaOCl/HEBP (+ H_2_O after final irrigation)	TotalFill HiFlow BC (+ Fluo-3)	Lateral condensation + temporary filling (Cavit)
Lemos et al. (2022) [43]	Distobuccal root of maxillary molar (30: 10 × 3)	Final irrigation ^3^ K10, K25, K40 with VDW Silver motor	G-1: 2.5% NaOCl (PUI); G-2: 0.9% saline (PUI); G-3: H_2_O (PUI)	Sealer Plus BC (+Fluo-3)	Gutta-percha single cone
Mahdi et Talabani (2025) [44]	Mandibular single-rooted premolar (44: 11 × 4)	Irrigant activation ^2^ WaveOne Gold up to medium size (35/06)	5.25% NaOCl + H_2_O; G-1: conventional, G-2: heat activation, G-3: diode laser, G-4: XP-Endo Finisher	Bio-C (+rhodamine B)	Gutta-percha single cone
Martinho et al. (2020) [45]	Single-rooted tooth (29: 10 + 10 + 9)	Final irrigation ^3^ ProTaper (F3)	G-1: 17% EDTA + 3% NaOCl; G-2: 17% EDTA + 2% CHX; G-3: 17% EDTA + saline	MTA Fillapex (+rhodamine B)	Lateral condensation with gutta-percha
Mehdat et al. (2022) [46]	Mandibular single-rooted premolar (30: 10 × 3)	Final irrigation ^3^ K15 + ProTaper Next (X4)	G-1: 0.2% CNPs; G-2: 0.2% CNPs + 17% EDTA; G-3: 17% EDTA	A: SureSeal Root BC; B: AH Plus	Single cone
Öztürk et al. (2025) [47]	Maxillary single-rooted incisor (180: 15 × 3 × 4)	Final irrigation ^3^ K10 + ProTaper Universal (F4) + H_2_O after final irrigation	G-1: 17% EDTA; G-2: 9% HEBP; G-3: 1% peracetic acid; G-4: H_2_O	BioSerra (+rhodamine B)	Gutta-percha single cone
Razmi et al. (2016) [48]	Single-rooted premolars (18: 9 × 2)	Irrigation ^3^//sealer ^1^ K10 + ProTaper (F3)	G-1: 2% CHX; G-2: 5.25% NaOCl	AH Plus; Adseal; EndoSequence	Lateral condensation
Saghebi et al. (2025) [49]	Mandibular premolar with one canal (40: 10 × 4)	Irrigant activation ^2^ K10 + ProTaper (F3)	5.25% NaOCl + saline; G-1: no activation; G-2: ultrasonic; G-3: XP-Endo Finisher; G-4: diode laser	MTA Fillapex	Gutta-percha single cone
Shekhar et al. (2025) [50]	Mandibular premolar with one canal (32: 8 × 4)	Final irrigation ^3^ K15 + ProTaper (F3)	G-1: saline; G-2: 17% EDTA; G-3: 10% citric acid; G-4: 7% MA	BioRoot RCS (+rhodamine B)	Ultrasonic condensation with gutta-percha
Talreja et al. (2023) [51]	Single-rooted with one canal (42: 7 × 6)	Irrigant activation ^2^//sealer ^1^ K10 + ProTaper Universal (F3)	17% EDTA + 5% NaOCl + H_2_O (1 min); A-B: no activation; B-C: PUI; E-F: YAG laser	AH Sealer or CS sealer	Master gutta-percha cone F3
Vivek et al. (2025) [52]	Single-rooted (80: 40 × 2)	Sealer activation ProTaper (F3)	5.25% NaOCl + 17% EDTA + saline; G-U: ultrasonic; G-L: lentulo spiral; G-G: gutta-percha + US; G-C: control	Bio-C; AH Plus	Gutta-percha single cone
Zand et al. (2019) [53]	Single-rooted (40: 10 × 4)	Irrigant activation ^2^ K10 + BioRace (#40)	17% EDTA + 5.25% NaOCl; G-1: conventional; G-2: PUI; G-3: Er:YAG laser; G-4: XP-Endo Finisher	The Sure Seal Root BC	Gutta-percha single cone

^1^: grouping according to sealers/CSSs; ^2^: grouping according to irrigant activation; ^3^: grouping according to final irrigation; ^4^: grouping according to chelating agent.

**Table 4:** Study results.

Author (Year)	Penetration Measurement	Results
Ackay et al. (2016) [33]	Coronal, middle and apical thirds. CLSM (10×) + ImageJ. Tubular penetration area ^1^	iRoot SP > other sealers (*p* < 0.001) (1). PIPS and PUI > CI (*p* < 0.001) (1). Coronal and middle > other thirds (*p* < 0.001) (1)
Alim Uysal et al. (2021) [34]	Coronal, middle and apical thirds. CLSM + ImageJ. Maximum penetration depth ^2^. Mean penetration Depth ^3^	MA group > others in apical (*p* = 0.013) (3). Coronal > middle and apical in all irrigation groups (*p* < 0.001) (3). EndoSequence BC > other sealers (2). Minimum mean depth in apical, maximum in coronal (*p* < 0.001)
Alnoury et al. (2024) [35]	Middle third. PBS test (Push-Out Bond Strength) ^4^	Apple vinegar > other groups with EndoSequence BC (*p* < 0.05) (4). EDTA > others with Cerafill BC (4)
Ates et al. (2021) [36]	At 2 mm and 5 mm from apex. CLSM + ImageJ. TPSP ^3^ (total penetration %), SPA ^1^ (penetration area), MSPD ^2^ (maximum depth)	XPF (*p* = 0.004) and EA (*p* = 0.018) > laser at 5 mm (2). XPF > laser (*p* = 0.004) and CEN (*p* = 0.0001) at 5 mm (3). EA > laser (*p* = 0.035) at 5 mm (3). XPF > CEN and laser (*p* < 0.05) (1). EA > laser (*p* < 0.05) (1)
Aydin et al. (2018) [37]	At 3 and 5 mm. CLSM + ImageJ. Penetration depth ^3^, area ^1^, percentage ^5^	5 mm > 3 mm (*p* < 0.05) in all groups (3,1,5). CNPs < EDTA and QMix (*p* < 0.05) (3,1,5)
Bogari et al. (2022) [38]	At 2 mm. SEM (×400). PBS test ^4^ and GP–dentin gap	Laser > conventional and ultrasonic (*p* < 0.05) (4). Laser < conventional and ultrasonic (Gap between GP-sealer and dentin; (*p* < 0,05))
El Hachem et al. (2018) [39]	At 1 mm and 5 mm. CLSM + ImageJ. Max % ^2^, mean % ^3^, circumference % ^5^	5 mm > 1 mm for CN and EA (2,3 (*p* < 0.001)). 1 mm > 5 mm for CN (5)
Gawdat and Bedier (2022) [34]	At 5 mm. CLSM + LSM Image Examiner. Max depth ^2^, coverage % ^5^, area ^1^	G2 > G3 > G1 (1,5,2 (*p* < 0.05)). G2 (Dual: HEBP), G3 (NaOCl + EDTA), G1 (NaOCl)
Gupta et al. (2024) [42]	At 3–4–5 mm. CLSM (×10). Max depth ^2^	G2 (HEBP) > G3 (citric acid) > G1 (control) (*p* < 0.05) in all thirds
Hassan and Roshdy (2023) [42]	At 3–6–9 mm. CLSM. Max depth ^2^, percentage ^5^	Coronal > middle > apical (2,5 (*p* < 0.001)). G2 > G1 in coronal third (2,5; *p* < 0.05); G1 > G2 in apical third (5; *p* < 0.015)
Lemos et al. (2022) [43]	At 2–3–6 mm. CLSM. Sealer penetration ^3^	No penetration observed in any group
Mahdi and Talabani (2025) [44]	At 6–9 mm. CLSM + ImageJ. Max ^2^, min ^5^, gap ^5^	XP-Endo Finisher: higher apical penetration and better adaptation in middle third (*p* < 0.05)
Martinho et al. (2020) [45]	Middle and apical. CLSM (10×). Percentage ^5^	Coronal > middle > apical (*p* < 0.05)
Mehdat et al. (2022) [46]	Silver staining + SEM. Apical sealing (nanoleakage)	SureSeal BC < AH Plus (*p* < 0.001). No differences between irrigants. AH Plus higher leakage with EDTA
Öztürk et al. (2025) [47]	At 2–5–8 mm. CLSM (×5) + ImageJ. Max ^2^, area ^1^, percentage ^5^	Coronal > middle > apical (2,1,5; *p* < 0.001). Control > others regardless of irrigation (1,5)
Razmi et al. (2016) [48]	Middle third. Push-out test ^4^. Failure mode	ADSEAL: no differences. AH Plus: moisture affects bond strength (dry > higher). EndoSequence: CHX lower strength than NaOCl
Saghebi et al. (2025) [49]	At 2–5–8 mm. SEM (×1000). Penetration ^3^	Laser > US in apical (3). Laser > others in middle (3). Ultra-X < G3 and G4 (*p* < 0.001), G3 < LÁSER (*p* < 0.001) in coronal
Shekhar et al. (2025) [50]	At 2–5–8 mm. SEM + image analysis. Mean depth ^3^	Coronal > middle > apical (*p* < 0.05). Differences in middle/apical between EDTA vs. CA/MA
Talreja et al. (2023) [51]	At 2–5–8 mm. CLSM + ImageJ. Area ^1^, mean ^3^	Laser > others in all thirds (1; *p* < 0.001). Apical < middle and coronal (3; *p* < 0.01)
Vivek et al. (2025) [52]	Micro-CT images (NRecon). Percentage ^5^	Activation systems > control (5; *p* = 0.001). BioC: ultrasonic > lentulo. AH Plus similar pattern
Zand et al. (2019) [53]	SEM analysis. Max Depth ^2^	G2 > G1 and G4 in apical. G2 highest in coronal (2)

^1^: total penetration area; ^2^: maximum penetration depth; ^3^: mean penetration depth; ^4^: PBS test; ^5^: other variables.

**Table 5:** Summary of the main trends observed for irrigation protocols and activation methods.

Category	Protocols or Methods Evaluated	Main Laboratory Trend	Main Limitations Affecting Interpretation
NaOCl- and EDTA-based protocols	NaOCl, EDTA, and NaOCl + EDTA sequences	Frequently investigated and commonly associated with favorable CSS penetration, particularly when smear layer removal was achieved	Protocols differed in concentration, sequence, contact time, final rinse, drying protocol, sealer type, and assessment method
Continuous chelation protocols	NaOCl + HEBP/DualRinse	Some studies reported favorable penetration outcomes compared with conventional chelation protocols	Limited number of studies; heterogeneous sealers, outcome variables, and root thirds assessed
Alternative chelating agents	Maleic acid, citric acid, glycolic acid, apple cider vinegar, QMix, chitosan nanoparticles	Some agents showed promising results in individual studies, particularly in the apical third	Evidence limited to few studies per irrigant; direct comparison across studies is not reliable
Chlorhexidine or saline/control protocols	CHX, saline, distilled water, or control protocols	Generally less consistent or less favorable penetration outcomes compared with smear layer-removing protocols	Often used as comparators; protocols and outcome measures varied substantially
Conventional needle irrigation	Non-activated irrigation protocols	Frequently showed lower penetration outcomes than activated protocols	Used with different irrigants, sealers, and evaluation methods
Passive ultrasonic irrigation	Ultrasonic activation of irrigants	Often improved penetration compared with conventional irrigation in laboratory conditions	Activation parameters, irrigant sequence, and assessment methods were not standardized
Laser-assisted activation	PIPS, Er: YAG, Er, Cr: YSGG, diode, or other laser systems	Some studies reported favorable penetration outcomes, but results were inconsistent compared with ultrasonic or rotary activation	Different laser systems and parameters were used; limited comparability
Sonic and rotary activation systems	EndoActivator, XP-Endo Finisher, heat activation, lentulo spiral, and related methods	Some systems improved penetration in individual studies	Evidence remains method-specific and heterogeneous; no technique can be considered superior
Overall interpretation	All irrigation and activation approaches	Final irrigation and activation may influence CSS penetration under laboratory conditions	Evidence is limited by in vitro design, methodological heterogeneity, inconsistent outcome measures, and lack of clinical endpoints

## Data Availability

No new data were created or analyzed in this study. Data sharing is not applicable to this article.

## Supplementary Materials

The following supporting information can be downloaded at: https://www.mdpi.com/article/10.3390/ma19122682/s1.
